# A Molecularly Imprinted Sol-Gel Electrochemical Sensor for Naloxone Determination

**DOI:** 10.3390/nano11030631

**Published:** 2021-03-03

**Authors:** Narges Shaabani, Nora W. C. Chan, Abebaw B. Jemere

**Affiliations:** 1 Nanotechnology Research Centre, National Research Council Canada, Edmonton, AB T6G 2M9, Canada; Narges.shaabani@nrc-cnrc.gc.ca; 2 Defence Research and Development Canada—Suffield Research Centre, Medicine Hat, AB T1A 8K6, Canada; nora.chan@drdc-rddc.gc.ca

**Keywords:** electrochemical detection, molecularly imprinted polymers, sol-gel, naloxone, nanomaterials, multiwall carbon nanotube

## Abstract

A molecularly imprinted sol-gel is reported for selective and sensitive electrochemical determination of the drug naloxone (NLX). The sensor was developed by combining molecular imprinting and sol-gel techniques and electrochemically grafting the sol solution onto a functionalized multiwall carbon nanotube modified indium-tin oxide (ITO) electrode. The sol-gel layer was obtained from acid catalyzed hydrolysis and condensation of a solution composed of triethoxyphenylsilane (TEPS) and tetraethoxysilane (TES). The fabrication, structure and properties of the sensing material were characterized via scanning electron microscopy, spectroscopy and electrochemical techniques. Parameters affecting the sensor’s performance were evaluated and optimized. A sensor fabricated under the optimized conditions responded linearly between 0.0 µM and 12 µM NLX, with a detection limit of 0.02 µM. The sensor also showed good run-to-run repeatability and batch-to-batch performance reproducibility with relative standard deviations (RSD) of 2.5–7.8% (n = 3) and 9.2% (n = 4), respectively. The developed sensor displayed excellent selectivity towards NLX compared to structurally similar compounds (codeine, fentanyl, naltrexone and noroxymorphone), and was successfully used to measure NLX in synthetic urine samples yielding recoveries greater than 88%.

## 1. Introduction

In the United States and Canada, rates of prescription opioid analgesic misuse have surged in the last decade [1,2,3]. Effective medication-assisted treatment has been recognized as a priority to minimize the rates and societal costs of opioid use disorder [2,3,4]. Naloxone (NLX) is a known opioid antagonist drug that rapidly reverses the effects of opioid/heroin overdose by binding to the opioid receptors in the central nervous system [5,6]. Due to its efficacy, a number of NLX products are approved for use in medical emergencies worldwide, with each product containing different NLX doses [5,6]. Although NLX is considered a safe medication at standard doses, higher doses of NLX can cause undesirable behavioural changes, hypo-and hypertension, arrhythmia, pulmonary edema, seizure and cognitive impairment [5,7]. Measurement of urine NLX levels in medication-assisted treatment for opioid use disorder is a practice followed to help detect misuse and diversion of NLX-based drugs by patients of opioid use disorder [8]. Existing analytical methods of NLX measurement are based on liquid chromatography coupled with ultraviolet (UV) or mass spectrometry (LC-UV or LC-MS) detectors [9,10,11]. These methods, however, are time consuming, expensive and only suitable for centralized laboratory analysis. Hence, rapid, sensitive and portable analytical platforms for on-site quantitative measurement of NLX are sought. Electrochemical analysis methods, on the other hand, are shown to have fast response times, sensitivity, require far less instrumentation and cost less than the aforementioned techniques, are easy to miniaturize, and are suited for real time on the spot measurements [12,13,14].

Electrochemical-sensing platforms employing molecularly imprinted polymers (MIPs) as recognition entities have attracted much attention in chemical and biological sensors preparations due to MIPs’ high selectivity, ease of preparation, low cost of fabrication, good chemical and physical stability as well as their reusability [15,16,17,18,19,20,21,22]. The preparation of MIPs entails polymerizing functional monomer in the presence of template molecule, which is typically the target analyte or molecule of similar size and chemical functionality. Upon template removal, 3D molecular recognition cavities that are complementary in size, shape and chemical functionality are formed. To the best of our knowledge only two MIP-based electrochemical sensors are reported for NLX determination [23,24]. These conventional MIP sensors used organic molecules (para-aminobenzoic acid [23] and para-phenylenediamine [24]) as functional monomers, producing similar performances. Even though conventional MIP-based sensing strategies have expanded the field of chemical and biological sensor applications, they suffer from limitations such as slow diffusion of analytes to and from the binding sites, long response times, template leaching and low detection limits and sensitivities [22,25,26,27,28]. The fabrication of MIPs using sol-gel techniques have been shown to improve performances of MIP sensors due to their inherent permeability, porosity, large surface area, and good physical rigidity [25,26,27,28,29,30,31,32,33,34,35,36,37]. Although a number of literature reports have been published on the development of MIP-based electrochemical sensor platforms for the detection of clinically relevant chemicals and biomarkers in spiked pre-treated biofluids or semi-synthetic plasma, there remain a number of challenges and drawbacks that ought to be overcome to produce sensor platforms with the needed specificity and sensitivity for clinical use [38,39].

In this report, an electrochemical sol-gel MIP sensor for electrochemical determination of NLX is reported for the first time. The sol-gel MIP sensor was fabricated using triethoxyphenylsilane (TEPS) as the functional monomer, tetraethoxysilane (TES) as the cross linker and pyrrole to provide electrical conductivity to the sensor. Since sol-gel materials are not electrically conductive, fabrication of sol-gel MIPs for electrical and electrochemical sensing applications require the inclusion of conductive materials such as conductive polymers, metal and metal oxide nanoparticles to the sol-gel networks [26,29,32,34,37,40,41,42,43]. These also allow for sol-gel MIPs to be electrodeposited onto electrode surfaces. In this work, a thin uniform imprinted film composed of TES, TEPS and pyrrole with NLX as the template was electrochemically deposited onto a functionalized multiwall carbon nanotube modified indium-tin oxide (fMWCNT/ITO) electrode. TEPS was utilized as functional monomer because of its phenyl group ability to interact with the aromatic ring of NLX through π–π interaction. The heterocyclic amine group of pyrrole is capable of forming hydrogen bond with the hydroxyl group of NLX, so pyrrole which is incorporated into the sol solution to increase the conductivity of the resulting sol-gel MIP can also play a role of additional functional monomer. The prepared pyrrole@sol-gelMIP/fMWCNT/ITO sensor displayed recognition of NLX with good reproducibility, selectivity and stability against structurally analogous compounds. The sensor was also used to quantitate NLX in artificial urine sample, producing high recovery.

## 2. Experimental

### 2.1. Chemicals and Materials

Naloxone (NLX) hydrochloride dihydrate, 1 mg/mL naltrexone (NAL) in methanol, 1 mg/mL noroxymorphone (NOMO) in methanol, 1 mg/mL fentanyl (FEN) in methanol, 1 mg/mL codeine (CO) in methanol, tetraethoxysilane (TES) (>99.0%), triethoxyphenylsilane (TEPS) (98.0%), pyrrole (>98.0%), high-performance liquid chromatography (HPLC) grade trifluoroacetic acid (TFA) (>99.0%), HPLC grade dimethylformamide (DMF) (>99.9%), reagent grade lithium perchlorate (>95.0%), reagentplus^®^ grade potassium ferricyanide (~99%), reagentplus^®^ grade potassium ferrocyanide (98.5%), reagentplus^®^ grade potassium nitrate (>99%), reagent grade hydrochloric acid (37%), reagent grade sulfuric acid (95.0–98.0%), 30% hydrogen peroxide, HPLC grade acetone, HPLC grade methanol, 28% ammonium hydroxide, 70% nitric acid, HPLC grade ethanol, phosphate-buffered saline (PBS) tablets, and MWCNTs (>95%, OD 6–13 nm, L 2.5–20 µm) were obtained from Sigma-Aldrich (Oakville, ON, Canada). Artificial urine was purchased from Pickering Laboratories (Mountain View, CA, USA). Indium tin oxide (ITO) coated glass slide (25 × 75 × 1.1 mm, Rs 30–60 Ω/square) was purchased from Delta Technologies (Loveland, CO, USA). Deionized (DI) water ensuring a resistivity of 18 MΩ.cm (Milli-Q UV Plus Ultra-Pure Millipore System) was used to prepare aqueous solutions and to rinse electrodes. PBS buffer was prepared to 50 mM pH 7.4 by dissolving PBS tablets in DI water. 50 mM NLX stock solutions were prepared in DI water and stored at 4 °C. Daily NLX working solutions were prepared by diluting the stock solution in PBS. Pyrrole was distilled and stored under N_2_ in a dark bottle at 4 °C prior to use.

### 2.2. Preparation of Polypyrrole@solgel Molecularly Imprinted Polymer (MIP) Sensor Preparation

**Scheme 1:**
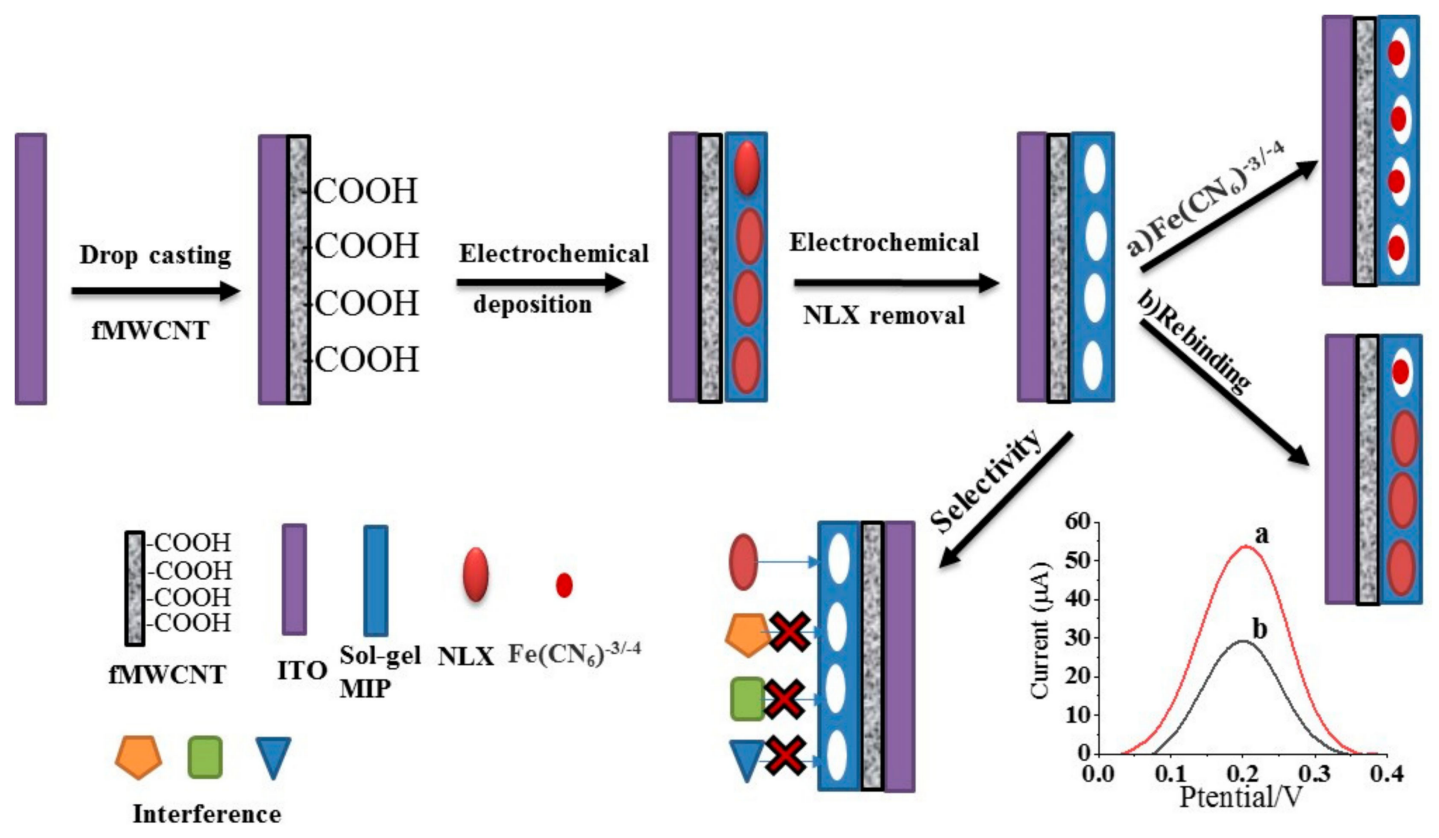
Illustration of stepwise fabrication of pyrrole@sol-gel MIP sensor and the NLX detection scheme.

Scheme 1 shows the stepwise fabrication of the sol-gel MIP sensor. Prior to modifying the ITO electrode with fMWCNT, pristine MWCNTs were oxidized using HNO_3_ according to a previously published protocol [41] to generate carboxylic acid functional groups (-COOH) that would promote dispersion and stability of MWCNTs in aqueous medium. 0.5 g of MWCNTs was added to 60 mL of conc. HNO_3_ and sonicated for 30 min. The mixture was then refluxed at 85 °C under magnetic stirring for 12 h. Once the mixture was cooled to room temperature, it was filtered using a polycarbonate membrane (0.22 µm) and washed with DI water until the pH of the filtrate reached 7. The solid material was dried for 3 days in a freeze-dryer, resulting in functionalized MWCNT (fMWCNT).

ITO-coated glass substrates were sonicated in a 10% detergent solution for 10 min, rinsed and sonicated with DI water, ethanol, and acetone for 10 min each. The cleaned electrodes were finally rinsed with DI water and dried in an N_2_ stream prior to activation using H_2_O_2_ (30%)/NH_4_OH (30%)/H_2_O (1:1:5, v/v/v) for 30 min to obtain surface hydroxyl (–OH) groups. The electrodes were finally washed with copious amounts of DI water and dried using a stream of N_2_. A geometrical area of 0.28 cm^2^ was defined in each electrode using Kapton tape, and modified with fMWCNT. To modify, 20 µL of 1 mg suspension of fMWCNT prepared in 1 mL of DMF/water (50:50, v/v) was pipetted onto the ITO electrodes and allowed to dry at 60 °C in an oven overnight, and the resulting fMWCNT/ITO electrode was thoroughly washed with DI water.

To form the imprinted sensor film (polypyrrole@solgelMIP/fMWCNT/ITO), 0.15 mL (0.8 mmol) TEPS, 0.15 mL of (0.7 mmol) TES, 0.01 mL of (0.08 mmol) TFA, 1.1 mL of C_2_H_5_OH, 0.7 mL of H_2_O were thoroughly mixed for 1 h at room temperature to yield the initial sol. To this sol solution, 0.0034 gm NLX (yielding 5.0 mM) was added and magnetically stirred for 1 h to prepare the imprinted sol. Then, 50 μL of pyrrole (0.36 mM final concentration) and 5.0 mg of LiClO_4_ were added and sonicated for 10 min. fMWCNT/ITO electrodes were then immersed in this polymerization solution, which was degassed using N_2_ gas, and cyclic voltammetry (CV) was carried out in the potential window of −0.8 V to +0.4 V (versus Ag/AgCl) at a scan rate of 50 mV s^−1^ for 10 cycles to fabricate the pyrrole@sol-gelMIP/fMWCNTs/ITO sensor. The prepared pyrrole@sol-gelMIP/fMWCNTs/ITO electrodes were then dried at room temperature for 2 h, and finally put in an oven at 60 °C overnight. (This step is important to obtain a crack-free imprinted surface.) A control non-imprinted sol-gel polymer (pyrrole@sol-gelNIP was also prepared on the fMWCNT/ITO electrode under the same experimental conditions but in the absence of NLX in the polymerization solution. Extraction of the imprinted template NLX molecules from the polymer film was achieved by undergoing CV in 0.5 M H_2_SO_4_ in a potential window of 0.2–1.2 V for 10 cycles at a scan rate of 50 mV s^−1^.

Zeta potential values of MWCNT and fMWCNT were obtained using a Zetasizer Nano-ZS (Malvern Panalytical, St. Laurent, QC, Canada) by dispersing 0.05 mg of each sample in 1 mM KCl (pH 6.0), followed by sonication for 30 min. Fourier transform infrared (FTIR) measurements were conducted on 0.1% MWCNT and fMWCNT samples prepared onto KBr pills using a Carry 670 Series FTIR (Agilent Technologies, Penang, Malaysia), with 0.075 cm^−1^ spectral resolution to confirm the presence of functional groups on the surface of MWCNTs. fMWCNT and sol-gel MIP modified electrodes were characterized by scanning electron microscopy (SEM, Hitachi S-4800, Japan).

### 2.3. Electrochemical Measurements

All electrochemical measurements were carried out with PalmSens potentiostat-galvanostat controlled by PSTrace 5 software version 5.5.241(Randhoeve, Houten, The Netherlands), using a pyrrole@sol-gel MIP/fMWCNTs/ITO electrode as the working electrode, Ag/AgCl and Pt wire electrodes as the reference and the counter electrode, respectively. In order to perform CV and differential pulse voltammetry (DPV) measurements, the sol-gel MIP sensor was first incubated in a NLX containing PBS buffer at pH 7.4 for 5 min. Following washing of the sensor with buffer and drying it off under a gentle stream of N_2_, the sensor was placed in a 1 mM K_3_[Fe(CN)_6_]/K_4_[Fe(CN)_6_] solution containing 0.1 M KNO_3_ as a redox pair probe and electrochemical measurements were conducted. CVs were recorded over a potential range of −0.2 to 0.6 V at a scan rate of 50 mV s^−1^. DPV experiments were performed by scanning the potential from −0.2 to 0.4 V, using a scan rate of 50 mV s^−1^, pulse width of 50 ms, and pulse period of 100 mV at room temperature.

## 3. Results and Discussion

### 3.1. Preparation and Characterization of Naloxone (NLX)-Imprinted Sol-Gel Sensor

#### 3.1.1. Surface Modification of ITO Electrode

Scheme 1 shows the stepwise fabrication of the sol-gel NLX imprinted sensor. First, ITO electrode was modified with carboxylic acid functionalized MWCNT (fMWCNT) to enhance the specific surface area of the electrode for the formation of sol-gel MIP films and its electrical conductivity. The introduction of –COOH functional groups to MWCNT was verified by FTIR spectroscopy and zeta potential measurements. The FTIR spectra of both pristine and functionalized MWCNT show a major peak at ~3450 cm^−1^ (Supplementary Figure S1) which is ascribed to the stretching vibrations of surface –OH components and/or –OH of carboxyl groups in the samples and in sorbed water molecules [44,45]. The appearance of new peaks in the 1750–1550 cm^−1^ range for fMWCNT indicate the presence of C=O stretching bands that are characteristics of carboxyl functional groups and the successful oxidation of pristine MWCNT to form COOH-MWCNT (fMWCNT) [44,45]. The zeta potentials of MWCNT and fMWCNT, measured in 1 mM KCl (pH 6), were found to be −5.0 mV and −22.0 mV, respectively. The lower negative value for fMWCNT indicates the presence of more negative charges in fMWCNT which is ascribed to the introduction of carboxyl functional groups as revealed in the FTIR spectra. fMWCNT modified electrodes were then characterized by SEM and electrochemical techniques.

**Figure 1:**
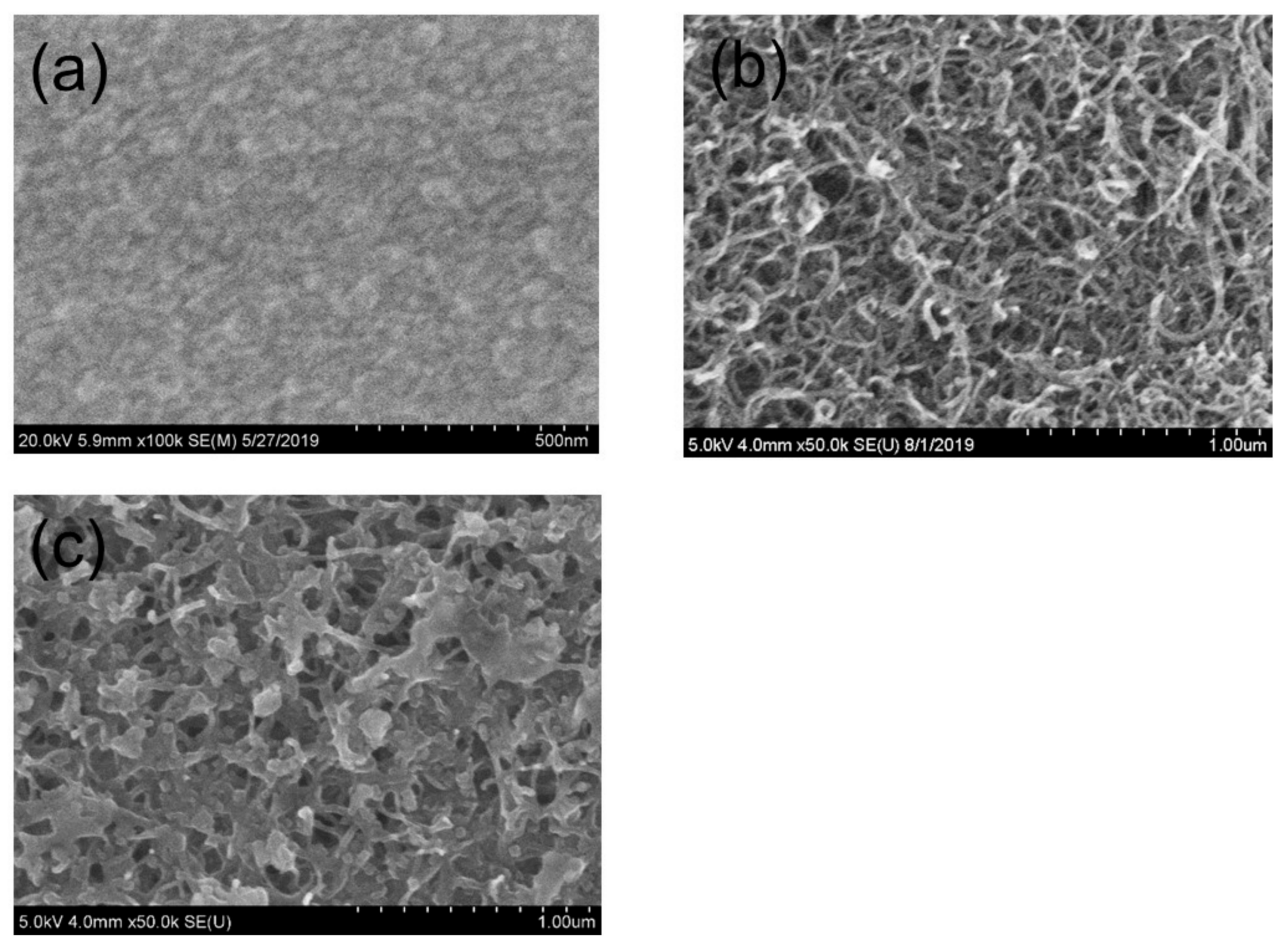
SEM images of (**a**) bare ITO, (**b**) fMWCNT/ITO and (**c**) pyrrole@sol-gel MIP/fMWCNT/IYO.

CVs of 1 mM [Fe(CN)_6_]^3−/4−^ on the two electrode types obtained at different scan rates are shown in Supplementary Figure S2. The peak currents (I_p_) of the redox probe increased linearly with the square root of the scan rate (µ^1/2^) for both electrodes, indicative of a diffusion-limited redox process. From the data shown in Supplementary Figure S2 and using the Randles–Sevcik equation [46], the electroactive surface area of the fMWCNT/ITO electrode was calculated to be 2.6 times higher than that of the bare ITO electrode, an electroactive surface area enhancement factor similar to reported values in the literature for fMWCNT modified electrodes [47,48]. SEM images shown in Figure 1a,b show clear differences in the morphologies of the bare ITO and the fMWCNT modified ITO electrodes. From the images it is evident that the fMWCNTs are uniformly distributed and formed a 3D network structure which enabled the fMWCNT/ITO electrode to have a higher specific surface area.

#### 3.1.2. Electropolymerization of Sol-Gel MIP Sensor

**Figure 2:**
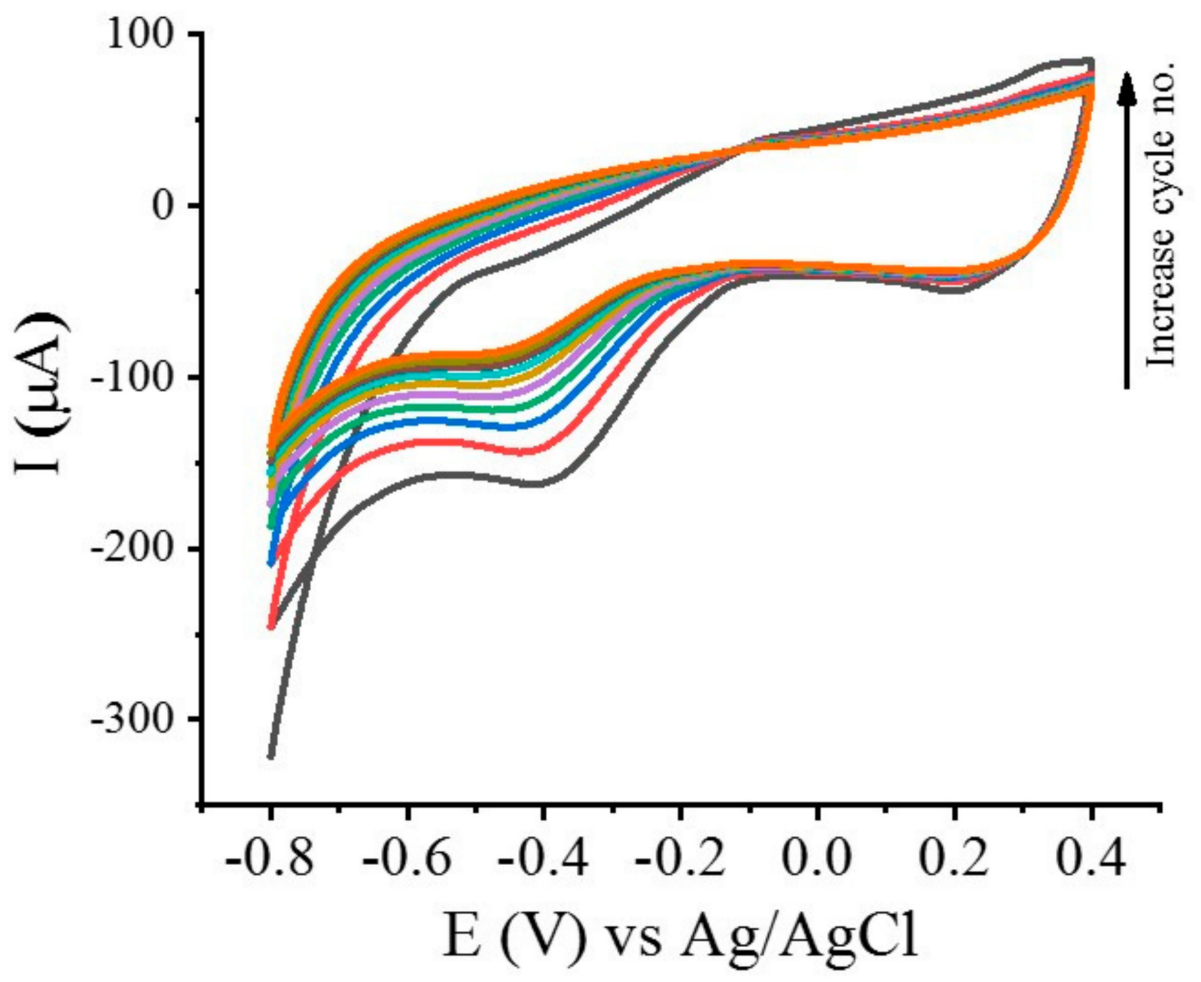
CV of the electropolymerization of sol-gel MIP on fMWCNT/ITO electrode at a scan rate of 50 mV/s.

Typical CV of the electropolymerization of the sol-gel MIP film on the fMWCNT/ITO electrode (Figure 2) shows that current decreased with increasing the number of electropolymerization cycles. This indicates continuous formation of an insulating sol-gel MIP film on the fMWCNT/ITO surface. No change in response current was observed after the 10th cycle, suggesting that the film had been formed completely. Thus, 10 cycles of electropolymerization were selected for the preparation of the sensor (pyrrole@sol-gelMIP/fMWCNT/ITO). No notable differences were observed in the CVs of the sensor pyrrole@sol-gelMIP/fMWCNT/ITO and the control pyrrole@sol-gelNIP/fMWCNT/ITO, indicating that the template NLX does not have any electrochemical redox property under the potential window of electrodeposition. Thus, the chemical structure of NLX is not affected by the imprinting process. Next, the prepared sensor was characterized by SEM, CV and DPV techniques.

Comparing the SEM images shown in Figure 1b,c, the size of the network structure observed for fMWCNT/ITO remarkably increased following the electropolymerization of pyrrole@sol-gelMIP on the electrode surface. The pyrrole@sol-gelMIP film appeared to wrap the fMWCNTs completely, and the surface of the network became much rougher, indicating successful electropolymerization and uniform deposition of the pyrrole@sol-gelMIP onto the fMWCNT/ITO electrode.

**Figure 3:**
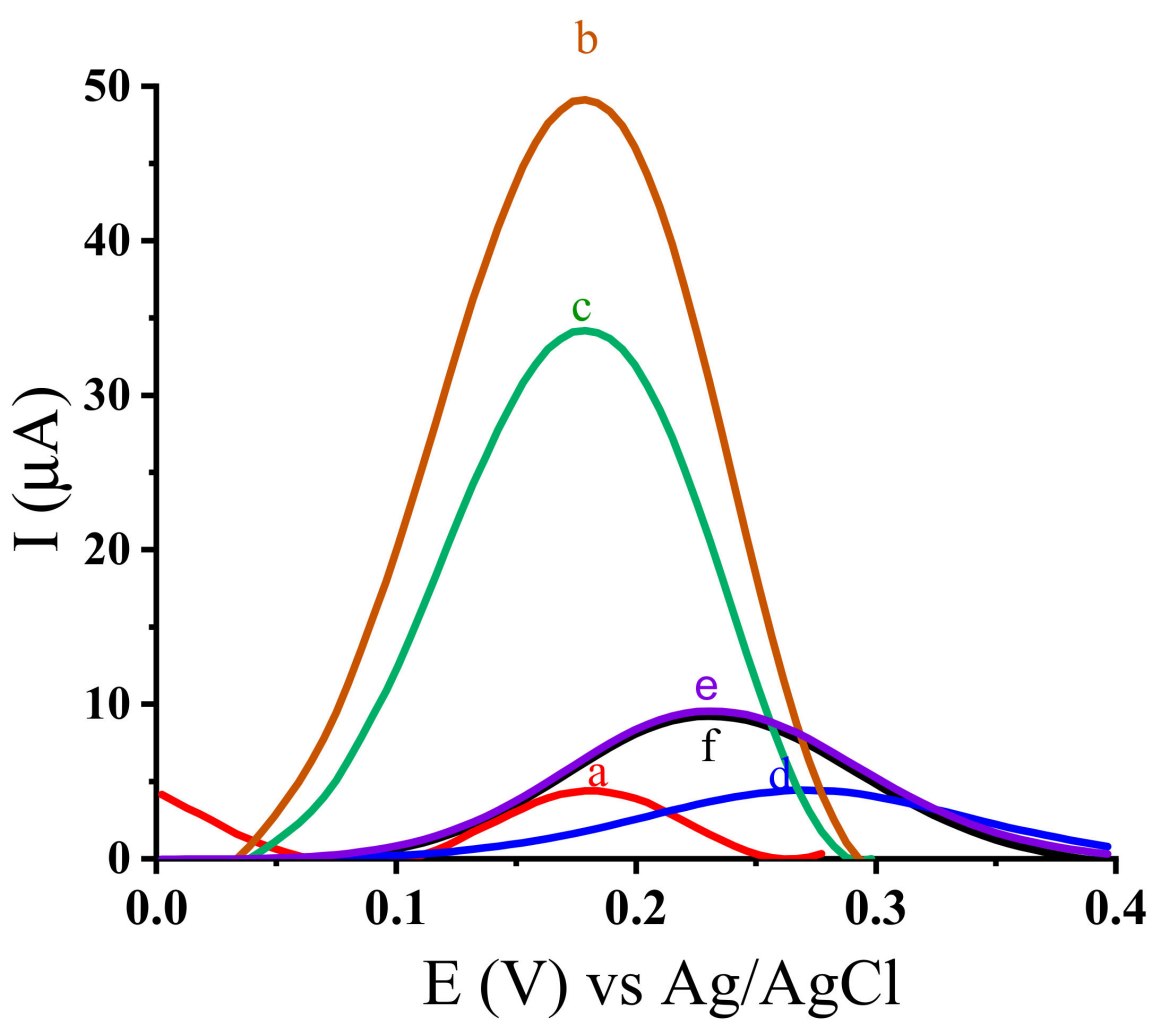
DPV of 1 mM [Fe(CN)_6_^3−/4−^] using pyrrole@sol-gelMIP/FMWCNT/ITO electrode (a) before NLX extraction, (b) after NLX extraction, (c) following 5 min incubation with 6 μM NLX. Curve (d) was obtained using as prepared pyrrole@sol-gelNIP/fMWCNT/ITO, curves (e & f) were obtained following NLX extraction and 5 min incubation with 6 μM NLX, respectively.

DPVs of 1 mM [Fe(CN)_6_]^3−/4−^ using pyrrole@sol-gelMIP/fMWCNT/ITO electrode prior to (Figure 3, curve a) and after template NLX removal (Figure 3, curve b), and followed by rebinding in 6 µM NLX (Figure 3, curve c) show the effects of template molecules in the sol-gel MIP. The synthesized sol-gel MIP matrix has a low peak current of ~4 µA (curve a). In this surface, the sol-gel MIP still contained the initial template molecules, thus access for the redox probe to the electrode surface is limited. Removal of the template NLX creates cavities in the sol-gel matrix which allows the transfer of electrons between the redox probe and the electrode surface, resulting in a significant increase in current to ~49 µA (curve b). Rebinding of 6 µM NLX to the sol-gel MIP matrix shows a decrease in the redox probe current (~29 µA, curve c) as a significant fraction of the cavities are filled with NLX blocking the redox probe from reaching the electrode surface.

Figure 3 (curves d–f) shows DPVs of 1 mM [Fe(CN)_6_]^3−/4−^ using the control sol-gel NIP (pyrrole@sol-gelNIP/fMWCNT/ITO) surface. DPV of the as-prepared sol-gel NIP produced ~4 µA, similar to that of the synthesized sol-gel MIP (curve a), although the peak position is ~0.1 V higher than that of the sol-gel MIP. When the sol-gel NIP was treated in similar fashion as the sol-gel MIP (i.e., template removal followed by NLX rebinding), there was only a slight effect on the DPV signals of the redox probe (~9 µA, curves e and f). These observations implied chemical physical entrapment of NLX in the pyrrole@sol-gelMIP/fMWCNT/ITO surface. CVs, shown in Supplementary Figure S3, of 1 mM [Fe(CN)_6_]^3−/4−^ obtained on bare ITO, fMWCNT/ITO and pyrrole@sol-gelMIP/fMWCNT/ITO support the above statement. The intensities of the redox currents observed for the fMWCNT/ITO electrode were higher than that of bare ITO, suggesting that the inclusion of fMWCNT, with its nanostructure and electrical property, increased the electrochemically active surface area of the modified electrode. Following the modification of the fMWCNT/ITO electrode with the sol-gel MIP, the redox current of [Fe(CN)_6_]^3−/4−^ noticeably decreased, indicating the formation of a polymeric insulating layer.

### 3.2. Optimization of the Sol-Gel MIP Sensor Preparation

For optimal sensor design, important sensor fabrication parameters such as the number of electropolymerization scan cycles, amount of monomer and crosslinker, pH of NLX solution, NLX extraction and incubation conditions were studied. The change in DPV current of 1 mM [Fe(CN)_6_^3−/4−^], (µI = I_o_ − I_NLX_), which was obtained by subtracting the current of the redox probe when the sensor was incubated in buffer alone (I_o_) from the current obtained following the sensor’s incubation with NLX (I_NLX_), was used to optimize the parameters. 

During electropolymerization of sol-gel MIP on an electrode surface, the number of polymerization cycles influences the thickness of the resulting polymer film, which in turn affects the sensors’ performance [28,32,37,43,49,50]. Thus, different pyrrole@sol-gelMIP/fMWCNT/ITO sensors were prepared from the imprinted sol solution described in the experimental section with CV polymerization cycles ranging from 2 to 20. Supplementary Figure S4a shows that the change in current increased with increasing the number of CV cycles, reaching a maximum at 10 cycles. Further increasing the cycle number resulted in decreased change in current, which is ascribed to the formation of thick sol-gel MIP layer that prevents complete extraction of template molecules [26,29,37,50]. Hence, 10 cycles of electropolymerization was chosen to fabricate the sol-gel MIP sensor.

In sol-gel MIP preparation, the amount of functional monomer and crosslinker in the polymerization solution influences the structure and recognition ability of the formed cavities [26,29,37]. Various pyrrole@sol-gelMIP/fMWCNT/ITO sensors were prepared by changing the amount of TEPS and TES in the range of 0.075–0.30 mL and varying the pyrrole concentration between 0.1–1.0 mM, while keeping the concentration of template NLX molecules at 5.0 mM. The best-performing sensor was found to be the one composed of 0.30 mL of silane (0.15 mL TEPS and 0.15 mL TES) and 0.35 mM pyrrole and this composition was chosen to make the sensor. Below these values of monomers (TEPS, TES and pyrrole), the responses of the sensors were low, which may be due to low number of imprinted sites formed. Conversely, when the concentrations were higher than the optimal values, very thick sol-gel MIP layers were formed, making complete extraction of the template NLX molecules difficult [28,37].

To examine the effect of pH of the NLX incubation solution on the rebinding of the analyte to the sol-gel MIP sensor, a pyrrole@sol-gelMIP/fMWCNT/ITO sensor was incubated with 12 µM NLX prepared in 50 mM PBS with varying pHs (ranging from 6.5 to 8.5) for 5 min. Supplementary Figure S4b shows that maximum sensor response was obtained at pH 7.4. NLX has an isoelectric point of 8.55 and it exists in four tautomeric forms in solution [51]. At pH 7.4, the cationic form predominates with a mole fraction of 79.6% [51], which may lead to optimal electrostatic and non-covalent interactions with the cavities formed in the sol-gel MIP. Thus, pH 7.4 was selected for subsequent experiments.

**Figure 4:**
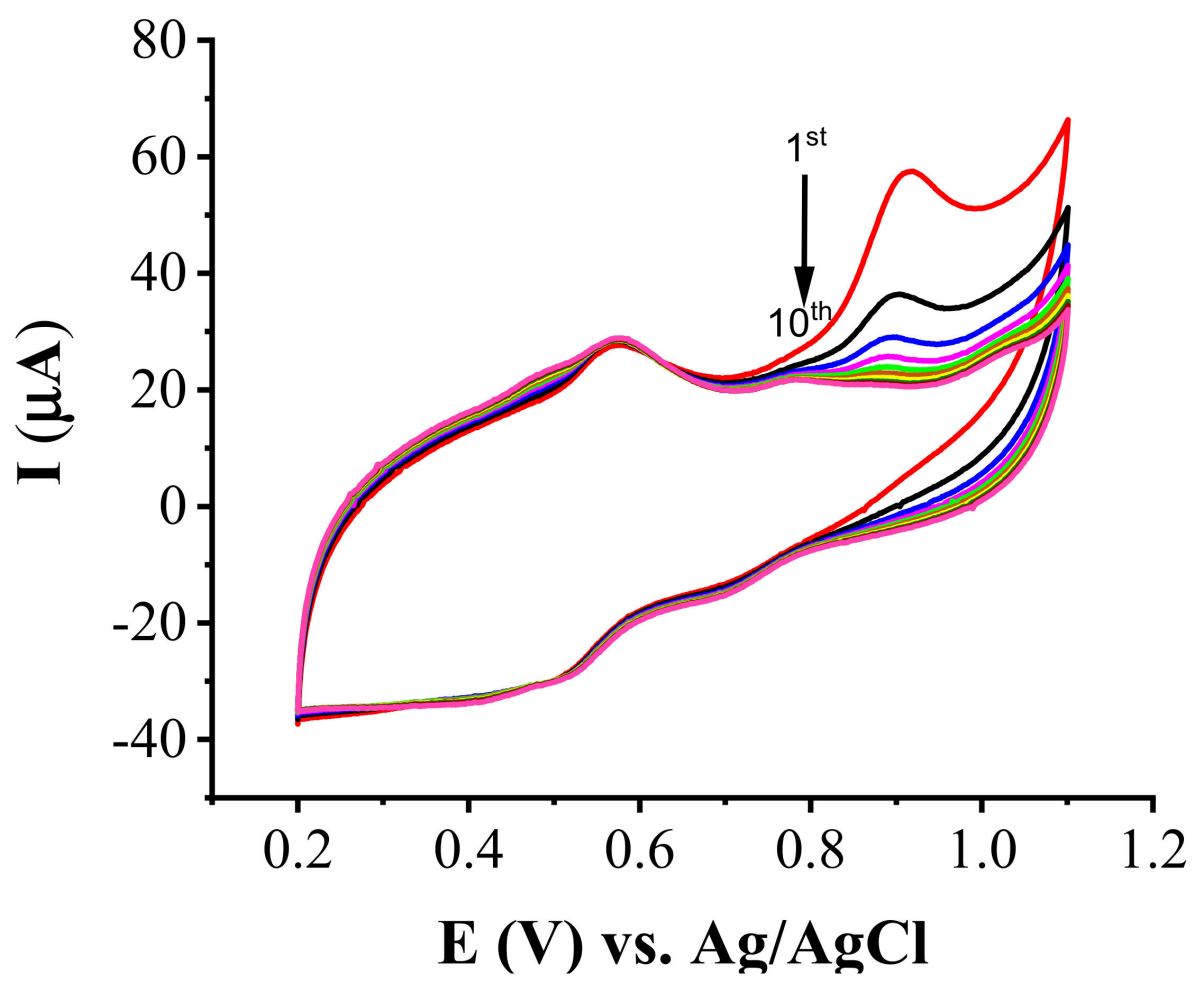
CV removal of entrapped NLX using 0.5 M H2SO4 at a scan rate of 50 mV/s.

Complete removal of entrapped template molecules from the sol-gel MIP matrix is necessary for the sensor’s performance and to be able to reuse the sensor after each NLX measurement. Solvent extraction is the most common and effective method of template removal in the development of sol-gel MIP sensors [18,52]. Here, two NLX removal methods (chemical and electrochemical methods) were examined. The chemical extraction method tested employed two solvent compositions, methanol/acetic acid (9/1, v/v) and acetonitrile/acetic acid (9/1, v/v). For the extraction method, extraction times were studied by soaking different sensors in methanol/acetic acid (9/1, v/v) and acetonitrile/acetic acid (9/1, v/v) solutions and measuring the DPV current of 1 mM [Fe(CN)_6_]^3−/4−^ in the resulting surfaces. Both solvents removed NLX from the sensors completely but required different lengths of time: MeOH/acetic acid required 70 min, acetonitrile/acetic acid needed over 16 h. The electrochemical technique used CV in 0.5 M H_2_SO_4_ in the potential range of 0.2–1.1 V. As shown in Figure 4, after the 5th cycle of CV, the peak at 0.91 V which was assigned to NLX oxidation, completely disappeared. Due to the short removal time (~3 min), the electrochemical method was used in subsequent studies.

The relationship between the rebinding time of NLX and current response was also optimized by incubating the sol-gel MIP sensor in 12 µM NLX solution prepared in 50 mM phosphate buffer (pH 7.4) for varying lengths of time. Supplementary Figure S4c shows that the response current (ΔI) increases with increasing the incubation time and reaches a plateau at 5 min. Therefore, an incubation time of 5 min was chosen for further studies. Both the NLX rebinding and electrochemical removal times reported here are significantly shorter than what has been reported for chemical extraction of NLX in MIP electrochemical NLX sensors (10–40 min) [23,24]. It seems likely that the fast response time of the sol-gel MIP electrochemical sensor results from the porous and permeable structure of the polymer film attained by the sol-gel technology.

### 3.3. Analytical Performance of the Sol-Gel MIP Sensor

**Figure 5:**
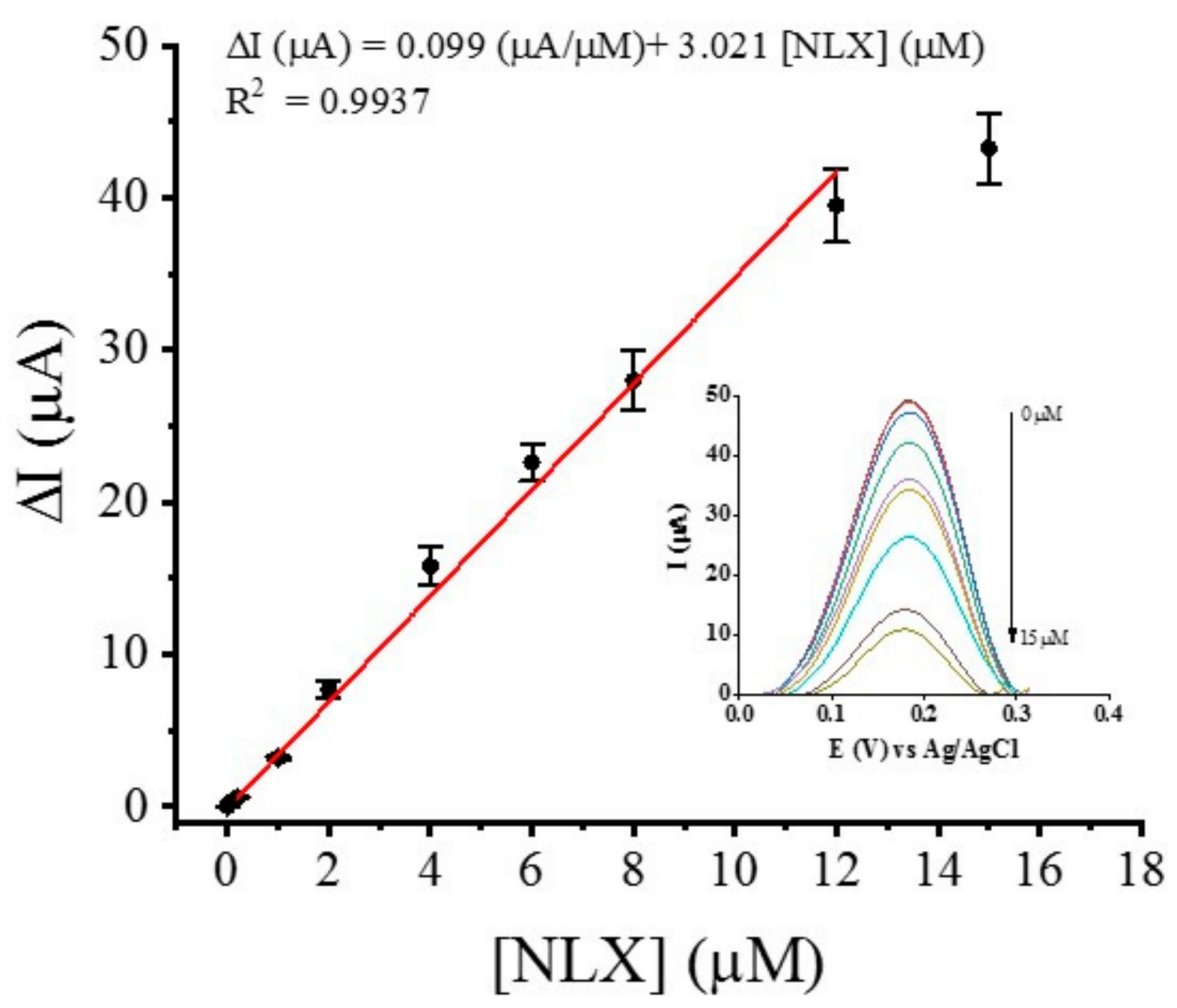
Calibration plot of the pyrrole@sol-gelMIP/fMWCNT/ITO sensor using different concentrations of NLX. The inset shows the DPV curves of the sensor using 1 mM [Fe(CN)_6_]^3−/4−^.

The analytical performance of a sensor, prepared under the optimized conditions, was examined using DPV of [Fe(CN)]_6_^3−/4−^. Following template removal and background current measurement, a sol-gel MIP sensor was incubated with varying concentrations of NLX for 5 min. The permeability of the sol-gel MIP surface to the redox probe decreases on the rebinding of NLX. This effect was used to quantify the analyte concentration [18,24,50]. As shown in Figure 5 inset, the [Fe(CN)_6_]^3−/4−^ current decreased as the NLX concentration was increased, indicating that a higher number of imprinted cavities were occupied by NLX molecules, and causing a greater extent of blocking of the redox probe. The plot of change in current as a function of NLX concentration yielded a straight line between 0.0 µM and 12 µM NLX (*R*^2^ = 0.9937), Figure 5. The slow rise in signal from 12 µM to 15 µM indicates approaching saturation of the binding cavities. Each data point represents an average of three measurements obtained using the same sensor, with the error bars showing the standard deviation of the three measurements. The limit of detection (LOD) of the sensor was calculated to be 0.02 µM NLX, based on 3 sd/slope, where sd was the standard deviation for the lowest NLX concentration tested. The present sensor has comparable linear range and an order of magnitude lower LOD compared to recently reported MIP NLX electrochemical sensors [23,24] and the HPLC-UV detection [53]. Similar LODs were reported for HPLC-UV [54] and HPLC-MS systems [55]. The amount of NLX in injectable and non-injectable preparations that are being used to treat opioid poisoning in North America and Europe are in the range of 0.4 mg mL^−1^ to 1 mg mL^−1^ (1.2 mM–3.0 mM) [5,6]. After oral or sublingual administration, NLX undergoes rapid and extensive hepatic metabolism, with serum half-life of 30–90 min [5], thus a substantial amount of NLX may not be available for routine urine analysis. For example, in a recent case study by Warrington et al. involving 1223 patients with an opioid disorder, the average amount of NLX found during urine test was 633.65 ng mL^−1^ (1.9 µM) [55]. Thus, developing sensors that are capable of detecting sub-micro molar concentration of NLX will be advantageous in both urine sample and pharmaceutical dosage analysis.

**Figure 6:**
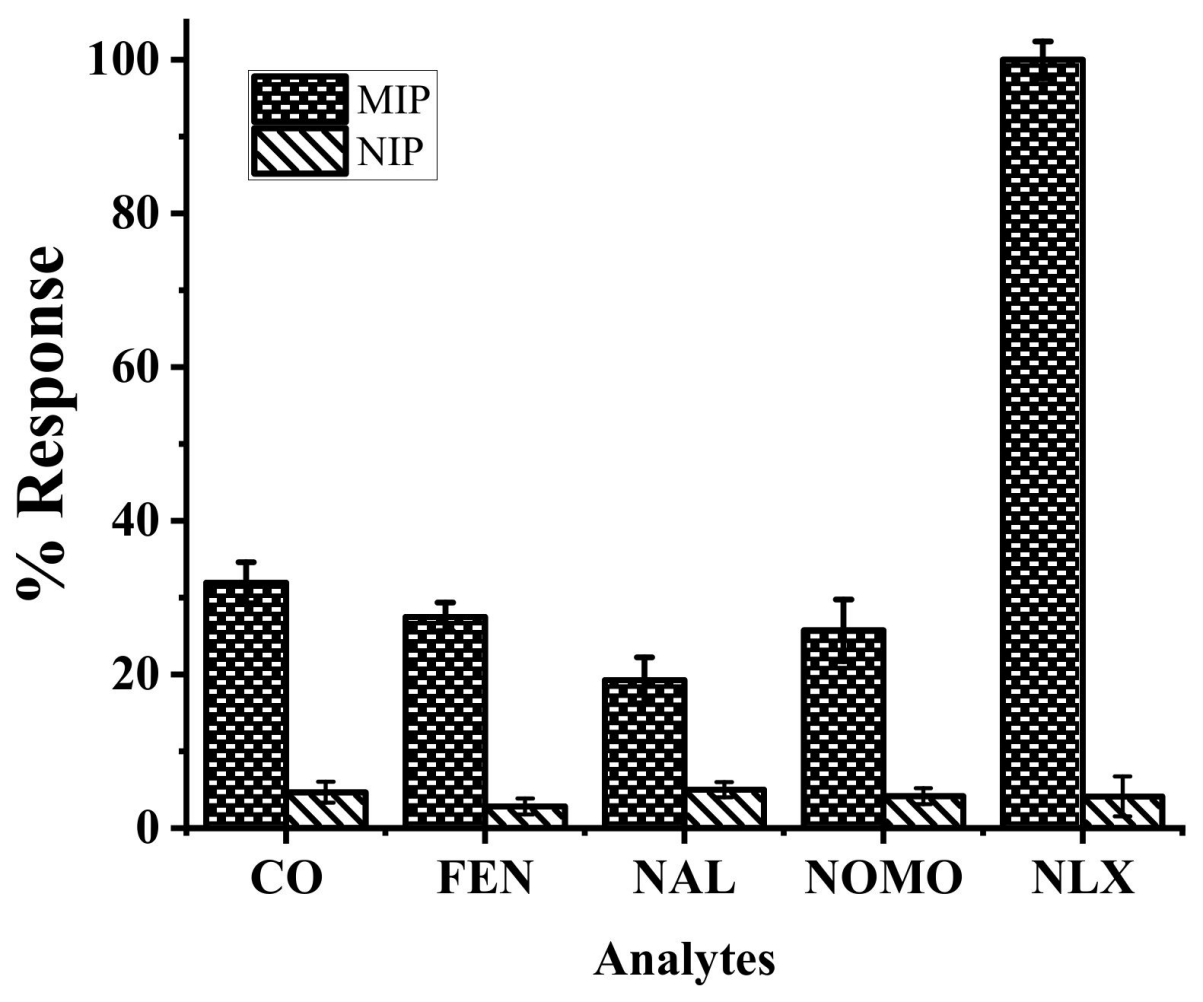
Percent current response of sol-gel MIP and sol-gel NIP modified fMWCNT/ITO sensors towards 12 mM NLX and its structural analogues. Error bars represent standard deviation of three independent measurements.

The selectivity of the sol-gel MIP sensor towards NLX was studied using structural analogue compounds of codeine (CO), fentanyl (FEN), naltrexone (NAL), and noroxymorpone (NOMO) (their chemical structures are given in Supplementary Figure S5) prepared individually at 12 µM. Figure 6 displays the response of the sol-gel MIP sensor towards NLX was 3–5 times higher than those of the non-target compounds, demonstrating that the sensor has higher recognition selectivity to NLX. The control non-imprinted sol-gel NIP accounted for only ≤5% of the total signal obtained for the sol-gel MIP sensor to all the tested compounds including NLX, suggesting the contribution of non-specific interaction to the sensor’s performance is small. The selective binding of NLX towards the sol-gel MIP sensor is driven by the discrete interactions between NLX functional groups and the pre-formed receptor cavities in the sol-gel MIP layer. The percent response of the sol-gel MIP sensor for NAL and NOMO (20–30%) is comparable to a reported value of ~20% by Shaabani et al. [24] (~20%), but better than the values (40–55%) reported by Lopes et al. [23] for the two molecules using MIP sensors.

The reproducibility of the sol-gel MIP sensor fabrication was examined by fabricating 4 independent sensors and interrogating them with a fixed concentration of NLX (4 µM), yielding a relative standard deviation (RSD) of 9.3%. Stability of the fabricated sensors was also evaluated by storing them for 14 days in a lab drawer under ambient conditions. The sensors maintained at least 92% of their original responses, demonstrating stability.

### 3.4. Detection of NLX in Urine Samples

The performance of the developed sol-gel MIP sensor was evaluated for NLX determination using artificial urine sample (Supplementary Table S1) by spike recovery experiments. NLX was spiked into diluted artificial urine (1:1 PBS) for final concentrations of 2, 7, and 10 µM. Table 1 displays the calculated recoveries using the equation from the calibration plot shown in Figure 5. The high recovery values demonstrate that the compounds in the artificial urine had only minor effect on the sensor performance and that the sensor has the potential for rapid measurement of NLX in urine.

## 4. Conclusions

This report describes the development of a rapid and selective molecularly imprinted sol-gel electrochemical sensor for the determination of NLX using an fMWCNT-modified ITO electrode. The developed sensor exhibited good performance in terms of selectivity, detection limit, response time, fabrication reproducibility and measurement repeatability. The excellent performance of the sol-gel MIP sensor can be ascribed to the porous structure of the polymer film attained by the sol–gel technology, and the high conductivity and increased surface area rendered by the fMWCNT layer, which lead to enhanced current transport and NLX immobilization sites per unit surface area of the electrode. Both NLX incubation and extraction times for the developed sol-gel MIP sensor are much shorter than those reported for the only two MIP NLX electrochemical sensors [23,24]. The detection limit of the pyrrole@sol-gelMIP/fMWCNT/ITO sensor also contrasts favourably to the two reported MIP NLX electrochemical sensors. The sensor retained 92% of its initial performance after 2 weeks of dry storage, and demonstrated excellent selectivity against structurally similar compounds (CO, FENT, NAL, and NOMO). The obtained high recovery of NLX in spiked urine samples indicates the sensor’s performance is not affected by matrices present in urine and the sensor could potentially be used in determining NLX concentrations in biological samples.

## Figures and Tables

**Table 1:** NLX concentration determination in spiked urine sample.

Spiked NLX Concentration(µM)	Measured Concentration (µM)	Standard Deviation (µM, n = 3)	%Recovery
2	1.770	0.0743	88.5
7	6.590	0.283	94.2
10	10.090	0.646	100.9

## Data Availability

Data are provided in the main text and as Supplementary Materials.

## Supplementary Materials

The following are available online at https://www.mdpi.com/2079-4991/11/3/631/s1, Figure S1: FTIR spectra of pristine and functionalized MWCNT, Figure S2: CV of 1 mM [Fe(CN)6]3^−^/4^−^ at different scan rates using (a) bare ITO electrode and (b) fMWCNT/ITO electrode, Figure S3: CV of 1 mM [Fe(CN)6]3^−^/4^−^ in (a) bare ITO, (b) fMWCNT/ITO, and (c) pyrrole@solgelMIP/fMWCNT/ITO obtained at a scan rate of 50 mV/s, Figure S4: Variation of the change in peak current of 1 mM [Fe(CN)6]3^−^/4^−^ in 0.1 KNO3 as a function of (a) number of electropolymerization cycle, (b) pH of the NLX incubation solution, and (c) NLX incubation time. The inset in (b) shows DPV of 1.0 mM K3 [Fe(CN)6]3^−^/4^−^ at pyrrole@sol-gel MIP/fMWCNT/ITO electrode before and after incubation in 12 μM NLX solution (50 mM PBS buffer, pH 7.4). For (a) three independent electrodes were prepared for the acquisition of each data, and for (b) and (c) three independent measurements were made on an pyrrole@sol-gelMIP/fMWCNT/ITO electrode, Figure S5: Chemical structures of the analyte naloxone, and the structurally similar molecules (codeine, fentanyl, naltrexone and noroxymorphone) tested in study, Table S1: List of ingredients in artificial urine.
