# Supplementary material for: A Molecularly Imprinted Sol-Gel Electrochemical Sensor for Naloxone Determination
Source: Nanomaterials (Basel). 2021 Mar 3;11(3):631. doi: 10.3390/nano11030631 (PMC8001154; doi:10.3390/nano11030631)
Supplement: Supplementary file 1 [file nanomaterials-11-00631-s001.pdf]

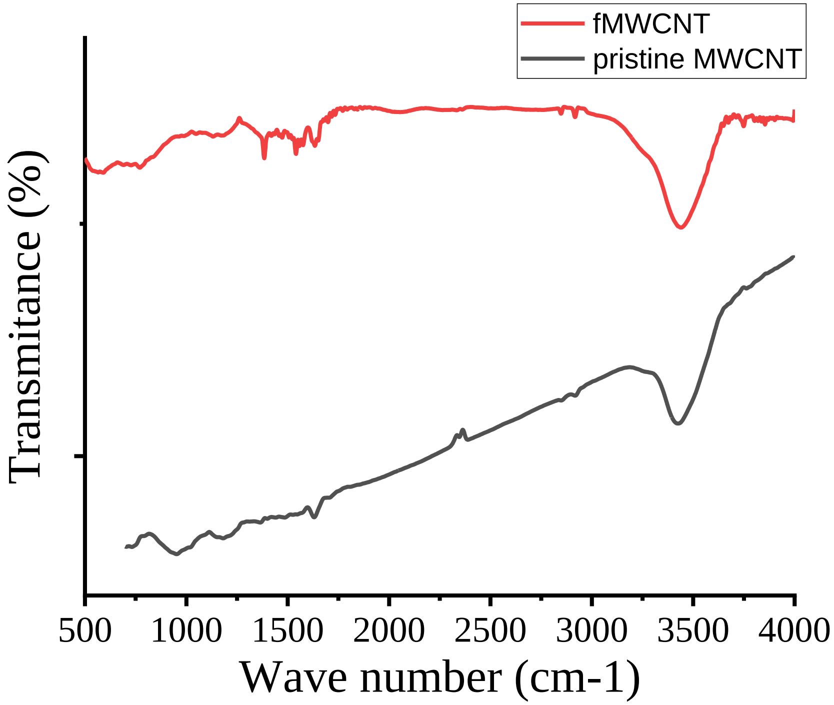

Supplementary Figure S1a. FTIR spectra of pristine and functionalized MWCNT.

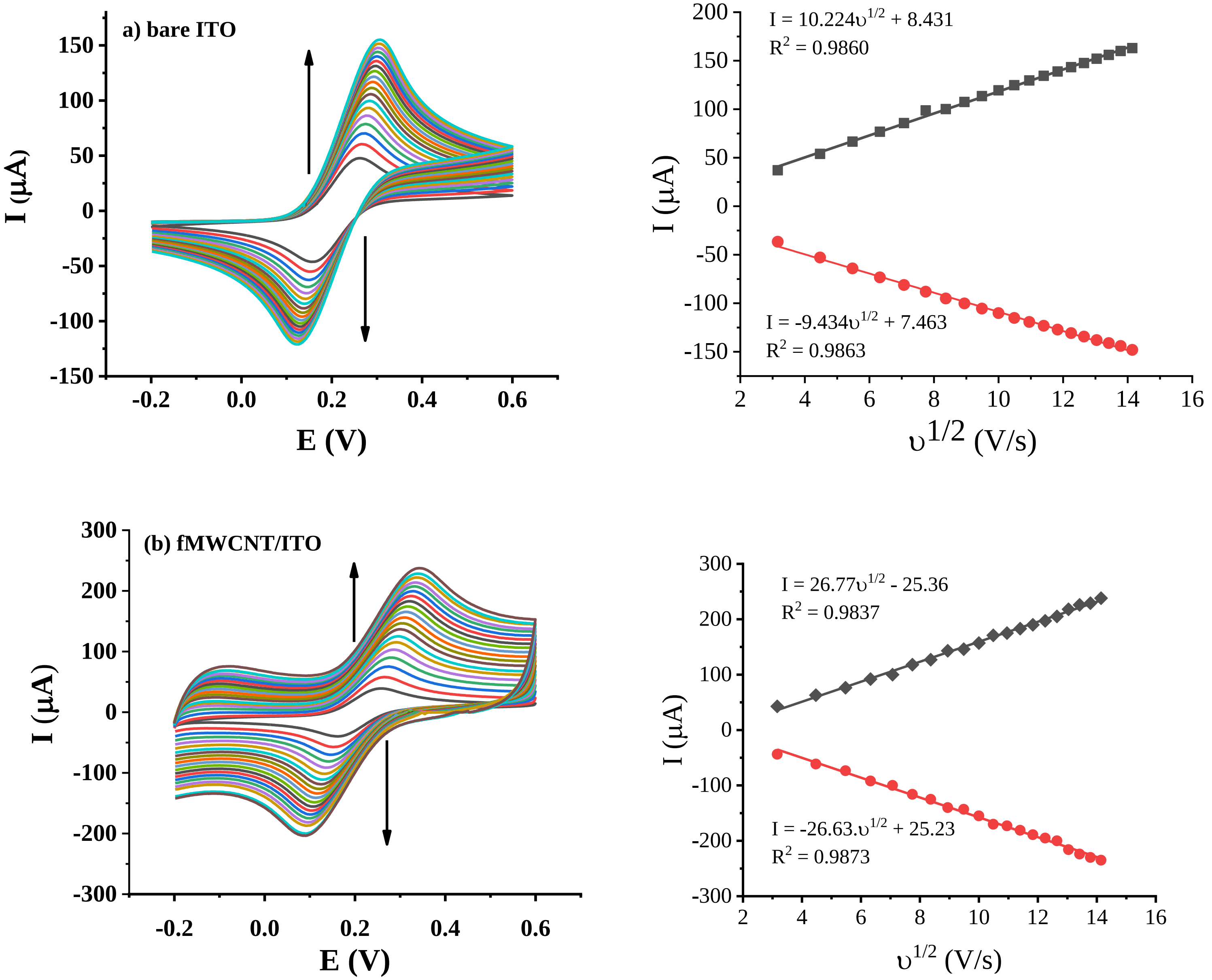

Supplementary Figure S2. CV of 1 mM [Fe(CN)6]3-/4- at different scan rates using (a) bare ITO electrode and (b) fMWCNT/ITO electrode.

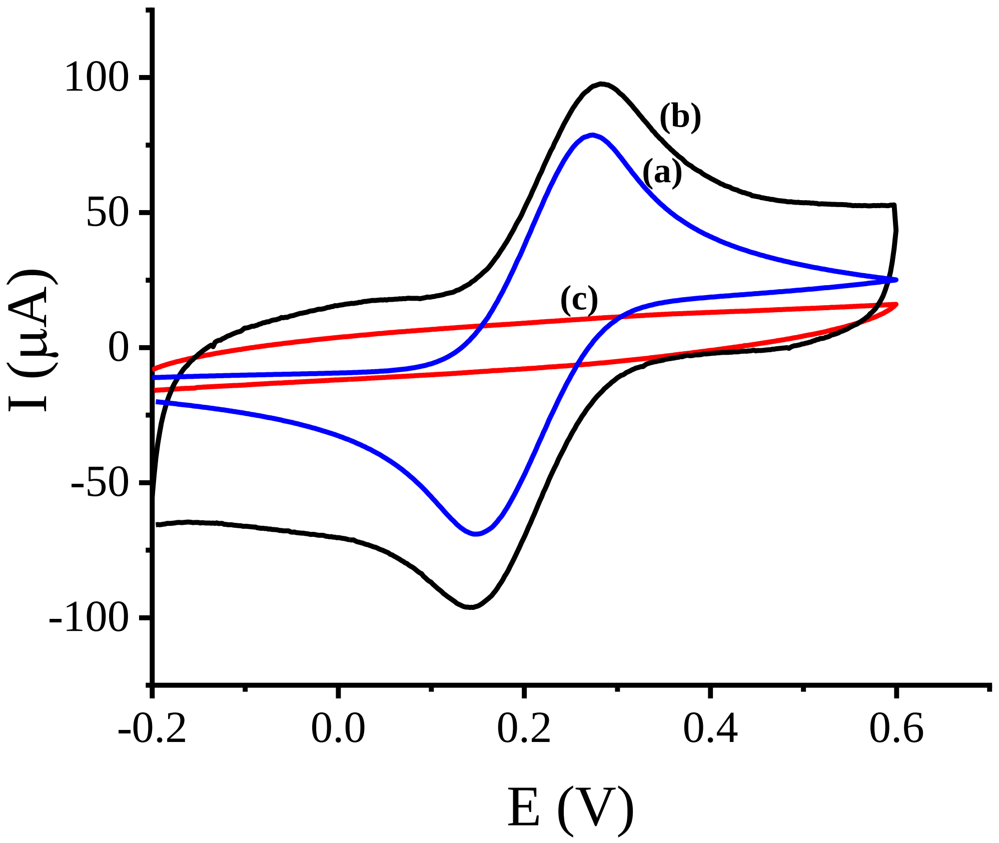

Supplementary Figure S3. CV of 1 mM $[Fe(CN)_6]^{3-/4-}$ in (a) bare ITO, (b) fMWCNT/ITO, and (c) pyrrole@solgelMIP/fMWCNT/ITO obtained at a scan rate of 50 mV/s.

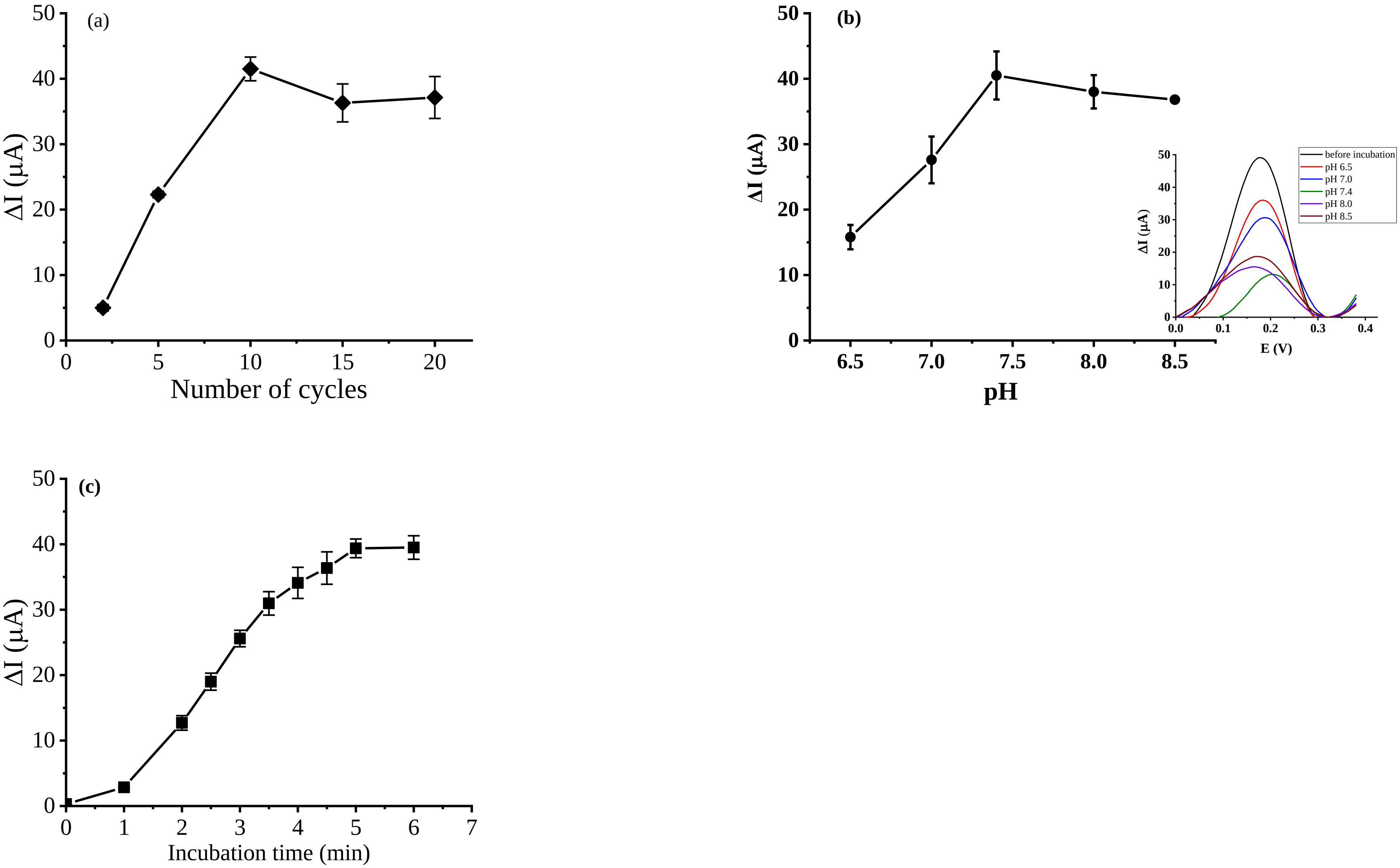

Supplementary Figure S4. Variation of the change in peak current of 1 mM $[Fe(CN)_6]^{3-/4-}$ in 0.1 KNO_3 as a function of (a) number of electropolymerization cycle, (b) pH of the NLX incubation solution, and (c) NLX incubation time. The inset in (b) shows DPV of 1.0 mM $K_3[Fe(CN)_6]^{-3/-4}$ at pyrrole@sol-gel MIP/fMWCNT/ITO electrode before and after incubation in 12 µM NLX solution (50 mM PBS buffer, pH 7.4). For (a) three independent electrodes were prepared for the acquisition of each data, and for (b) and (c) three independent measurements were made on an pyrrole@sol-gelMIP/fMWCNT/ITO electrode.

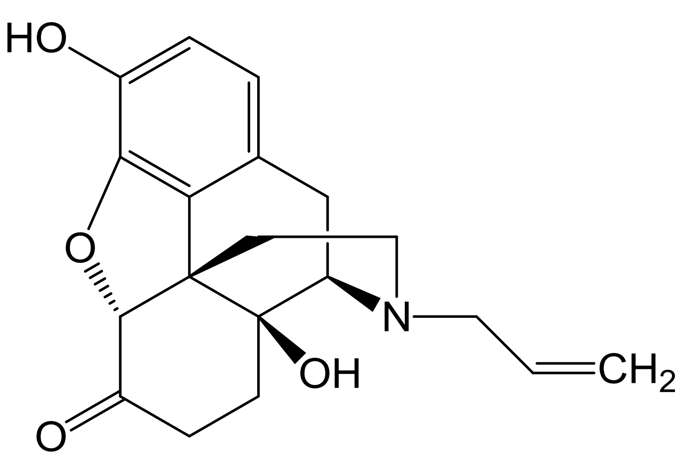

Naloxone

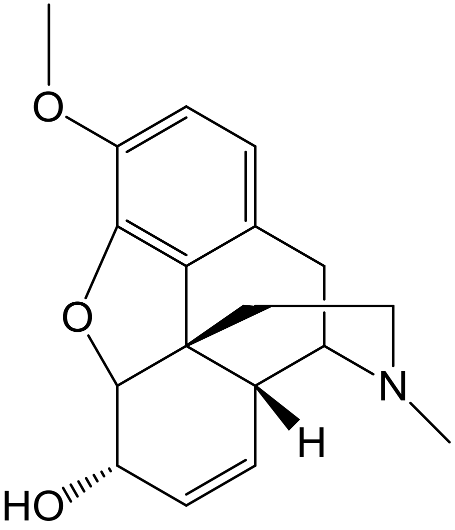

Codeine

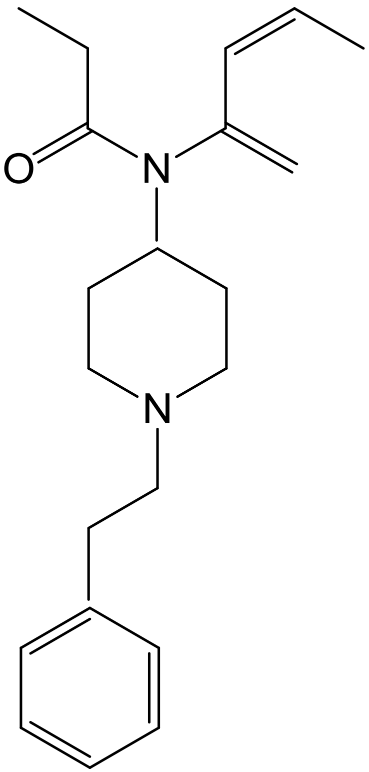

Fentanyl

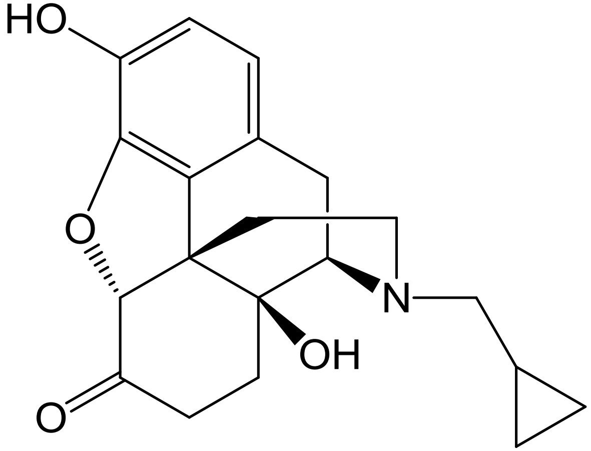

Naltrexone

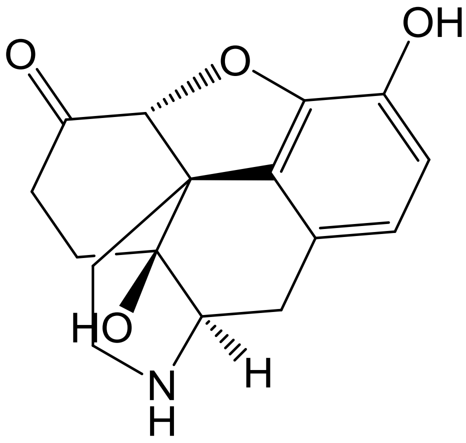

Noroxymorphone

Supplementary Figure S5. Chemical structures of the analyte naloxone, and the structurally similar molecules (codeine, fentanyl, naltrexone and noroxymorphone) tested in study.

Supplementary Table S1. List of ingredients in artificial urine

Ingredient	Concentration (g/L)
Urea	25
Sodium chloride	9
Disodium hydrogen orthophosphate, anhydrous	2.5
Potassium dihydrogen orthophosphate	2.5
Ammonium chloride	3
Creatinine	2
Sodium sulphite, hydrated	3
